# Supplementary material for: Impact of UV Exposure and Incidence of Merkel Cell Carcinoma Between 1990 and 2018 in Austria
Source: Cancers (Basel). 2025 Oct 20;17(20):3379. doi: 10.3390/cancers17203379 (PMC12562544; doi:10.3390/cancers17203379)
Supplement: Supplementary file 1 [file cancers-17-03379-s001.zip › cancers-3878929-supplementary.pdf]

Scheme Checklist: This completed STROBE (Strengthening the Reporting of Observational Studies in Epidemiology) checklist corresponds to the manuscript entitled "Impact of UV exposure and incidence of Merkel cell carcinoma between 1990 and 2018 in Austria." Each item is mapped to the relevant section and page of the manuscript.

Item	Recommendation	Reported in manuscript
1. Title and abstract	Indicate the study's design with a commonly used term in the title or abstract; provide an informative and balanced summary.	Title page, Abstract
2. Background/rationale	Explain the scientific background and rationale for the investigation being reported.	Introduction, page 3
3. Objectives	State specific objectives, including any prespecified hypotheses.	Abstract (Background), Introduction, pages 2+3
4. Study design	Present key elements of study design early in the paper.	Methods, page 4
5. Setting	Describe the setting, locations, and relevant dates, including periods of recruitment, exposure, follow-up, and data collection.	Methods (ANCR, UV data sources, years 1990–2018), pages 4–6
6. Participants	Give eligibility criteria, and the sources and methods of selection of participants.	Methods (ANCR patient inclusion criteria), pages 4–6
7. Variables	Clearly define all outcomes, exposures, predictors, potential confounders, and effect modifiers.	Methods (outcomes: MCC incidence, exposures: UV radiation; confounders: age, demographics), pages 4–6
8. Data sources/measurement	For each variable of interest, give sources of data and details of methods of assessment.	Methods (ANCR, UV data collection, coding), page 4–6
9. Bias	Describe any efforts to address potential sources of bias.	Discussion (possible bias: diagnostic awareness, registry limitations), pages 9–10
10. Study size	Explain how the study size was arrived at.	Methods (all cases of MCC in ANCR 1990–2018; cohort size 538), pages 4–6
11. Quantitative variables	Explain how quantitative variables were handled in the analyses.	Methods (incidence per 100.000; grouping of East vs. West), pages 4–6
12. Statistical methods	Describe all statistical methods, including those used to control for confounding.	Methods (statistical methods: t-test, Fisher's exact, correlation, Cox regression), page 6
13. Participants (Results)	Report numbers of individuals at each stage of study and reasons for non-participation.	Results (538 total patients, staging available for 308), pages 7–8
14. Descriptive data	Give characteristics of study participants and information on exposures and potential confounders.	Results (demographics, Table 1), pages 7–8
15. Outcome data	Report numbers of outcome events or summary measures.	Results (incidence rates, staging outcomes), pages 7–8
16. Main results	Give unadjusted and adjusted estimates, with precision (e.g., CI).	Results (correlations between incidence and UV; Figures 2–4), pages 7–8
17. Other analyses	Report other analyses done (subgroups, interactions, sensitivity analyses).	Results (subanalysis East vs. West, survival analysis), pages 7-8
18. Key results	Summarise key results with reference to study objectives.	Discussion, pages 9-10

19. Limitations	Discuss limitations of the study, sources of potential bias or imprecision.	Discussion (limitations: missing staging, unknown tumor site, registry completeness, aging population), page 10
20. Interpretation	Provide cautious interpretation of results, considering objectives, limitations, and other evidence.	Discussion (interpretation of incidence and UV link, comparison with literature), pages 10–11
21. Generalisability	Discuss generalisability of the study results.	Discussion (comparison with Scandinavian, Australian studies), pages 9–11
22. Funding	Give the source of funding and role of funders.	Funding statement, page 11
